# Long-term kinetics of *Salmonella* Typhimurium ATCC 14028 survival on peanuts and peanut confectionery products

**DOI:** 10.1371/journal.pone.0192457

**Published:** 2018-02-05

**Authors:** Maristela S. Nascimento, Joyce A. Carminati, Karen N. Morishita, Dionísio P. Amorim Neto, Hildete P. Pinheiro, Rafael P. Maia

**Affiliations:** 1 Department of Food Technology, School of Food Engineering, University of Campinas, Campinas, Brazil; 2 Department of Statistics, University of Campinas, Campinas, Brazil; University of Connecticut, UNITED STATES

## Abstract

Due to recent large outbreaks, peanuts have been considered a product of potential risk for *Salmonella*. Usually, peanut products show a low water activity (a_w_) and high fat content, which contribute to increasing the thermal resistance and survival of *Salmonella*. This study evaluated the long-term kinetics of *Salmonella* survival on different peanut products under storage at 28°C for 420 days. Samples of raw in-shell peanuts (a_w_ = 0.29), roasted peanuts (a_w_ = 0.39), unblanched peanut kernel (a_w_ = 0.54), peanut brittle (a_w_ = 0.30), *paçoca* (a_w_ = 0.40) and *pé-de-moça* (a_w_ = 0.68) were inoculated with *Salmonella* Typhimurium ATCC 14028 at two inoculum levels (3 and 6 log cfu/ g). The *Salmonella* behavior was influenced (p<0.05) by a_w_, lipid, carbohydrate and protein content. In most cases for both inoculum levels, the greatest reductions were seen after the first two weeks of storage, followed by a slower decline phase. The lowest reductions were verified in *paçoca* and roasted peanuts, with counts of 1.01 and 0.87 log cfu/ g at low inoculum level and 2.53 and 3.82 log cfu/ g at high inoculum level at the end of the storage time. The highest loss of viability was observed in *pé-de-moça*, with absence of *Salmonella* in 10-g after 180 days at low inoculum level. The Weibull model provided a suitable fit to the data (R^2^≥0.81), with δ value ranging from 0.06 to 49.75 days. Therefore, the results demonstrated that *Salmonella* survives longer in peanut products, beyond the shelf life (>420 days), especially in products with a_w_ around 0.40.

## Introduction

Traditionally low moisture foods have been considered as low risk for foodborne illness. Despite this, 9 salmonellosis outbreaks in peanuts or peanut products have been reported in the literature since 1994, with a total of 1,791 cases and 10 deaths [1–6]. The most recent outbreaks occurred in the USA, associated with peanut butter. The last report was in 2014 and had 6 cases [7].

Peanuts may become contaminated by *Salmonella* at any point throughout the supply chain. In primary production, soil, water, insects, birds, handlers and equipment are possible sources of contamination [8]. After heat treatment, the principal causes of cross-contamination are environmental processing and handling [9,10].

The shelf life of peanuts and peanut confectionary products range from six months to one year or more; it depends on the composition of the food and the storage condition. Although *Salmonella* does not grow in water activity (a_w_) below 0.94, it can often persist for long periods in low moisture foods and in a dry processing environment [11]. This characteristic may be one of the main factors that contribute to the occurrence of outbreaks in products with low a_w_.

It is known that osmotic stress caused by low a_w_ causes the activation of defense mechanisms in the microbial cell, such as the production of solutes (proline, trehalose and glutamine). The increase in the intracellular concentration of these compounds results in higher thermal and osmotic resistance [12]. Furthermore, the high fat content of peanuts also protects the pathogen against gastric acidity, resulting in a reduction dose-response curve with a low infectious dose. A very small number of *Salmonella* viable cells (0.5 to 5 MPN/g) was associated with an outbreak caused by *Salmonella* in almonds [13].

Therefore, for the adoption of preventive or control measures and for the implementation of risk management strategies, more studies are necessary to know the behavior of the microorganism in this product category. However, there is no data published on *Salmonella* survivability in peanut confectionary products, with the exception of peanut butter, and only one study on peanut kernels [14]. This is the first study that evaluated the survival of *S*. Typhimurium ATCC 14028 during long-term storage of different kinds of peanut based products.

## Material and methods

### Peanut-based products

Six types of peanut-based products obtained in a retail market located in Campinas-SP, Brazil (geographic coordinates 22°51’33.9” S and 47°06’22.7” W) were used: raw in-shell peanuts (a_w_ = 0.29), roasted peanuts (a_w_ = 0.39), unblanched peanut kernels (a_w_ = 0.54), peanut brittle (a_w_ = 0.30), *paçoca* (a_w_ = 0.40) and *pé-de-moça* (a_w_ = 0.68). All samples were previously tested for *Salmonella*.

The peanut confectionary products used for this study are traditionally consumed in Brazil. For peanut brittle manufacturing, peanuts are heated with a caramel sauce at 75°C for 1 h. *Paçoca* is a candy made of ground peanuts which is milled, mixed with sugar and salt, pressed and packed. *Pé-de-moça* is prepared with whole peanut kernels and condensed milk, which is heated at 110–120°C for 1 h.

### *Salmonella* strain and inoculum preparation

*S*. Typhimurium ATCC 14028 was used as the inoculum. It was stored at -80°C in trypticase soy broth (TSB, Difco, MD, USA) supplemented with 15% glycerol. For the inoculum preparation the strain was cultivated twice in trypticase soy broth (TSB) at 37°C for 18–24 h and then it was spread on plates of trypticase soy agar (TSA, Difco, MD, USA). After incubation at 37 ± 1°C for 24 h a cell culture loop was transferred to 0.85% saline tubes up to a turbidity of 5.0 MacFarland scale (10^9^ cells/ml). Then, decimal dilutions were performed in 0.1% peptone water (Difco, MD, USA) in order to obtain two different inoculum levels. Cell numbers in each inoculum suspension were determined by plating appropriate dilutions on TSA. The sample inoculation was performed as described in the following item.

### Inoculation of the samples

Initially, the samples were divided into 500 g portions and the confectionary products were crushed in order to obtain a more homogeneous inoculation. Then, the samples were inoculated by spraying with 0.2% (1 ml) of *Salmonella* suspension, plus 2% Tween 80 (Merck, DA, Germany) to reduce the surface tension [15]. The initial concentration of the inoculum in the samples was *ca*. 3 log cfu/ g (low inoculum level) and 6 log cfu/g (high inoculum level). After homogenization by hand for 2 min, the samples were transferred to aluminum screen trays and kept in a biosafety cabinet (Vecco, Brazil) for 10 to 20 min, with the purpose of ensuring maximum adherence of the inoculum and bring back the a_w_ closer to the original level [15]. Three 10g-portions from different locations in the same bag of each inoculated sample were taken to confirm the uniformity of the initial inoculum level. The inoculated samples were transferred to sterile bags. Peanut brittle and raw in-shell peanuts were stored in desiccators containing saturated solution of magnesium chloride (a_w_ = 0.32), and *paçoca* and roasted peanuts in desiccators containing saturated solution of potassium carbonate (a_w_ = 0.42) in order to keep the a_w_ stable throughout the storage. *Pé-de-moça*, unblanched peanut kernels and the desiccators containing the other products were stored in microbiological incubator at 28 ± 1°C for 420 days. Temperature and humidity of the microbiological incubator were monitored throughout the storage by a Testo 615 portable thermometer (Testo, Germany). For both experiments, the a_w_ value and the *Salmonella* count were determined after 0, 7, 14, 21, 28, 45, 60 days, and then every 30 days for up to 420 days. The experiments were replicated four times.

### Enumeration of *Salmonella*

The *Salmonella* enumeration was performed by the plate counting method. At each time point, portions of each inoculated sample were taken from different locations in the bag, to a total of 11 g, and homogenized by hand for 2 min with 99 ml of Buffered Peptone Water (BPW, Acumedia, MI, EUA). Then the samples were maintained at room temperature for 60 min to repair injured cells, but without favoring the growth. After, 10 ml of this first dilution were used to prepare subsequent serial dilutions in 0.1% peptone. Specific volume of each dilution was spread-plated onto Xylose Lysine Deoxycholate agar (XLD, Acumedia, MI, USA) and incubated at 37 ± 1°C for 24 h. When the counts were close to 10 cfu/g, 10 ml of the first dilution were pour-plated onto XLD (2.5 ml per plate) to improve the limit of detection (1 cfu/g). Presumptive-positive colonies were subjected to confirmation by biochemical and serological tests [16]. The results were expressed in log of colony forming unit (cfu) per gram. The remaining volume of the first dilution (100 ml) was also incubated at 37 ± 1°C for 24 h (pre-enrichment step). When counts decreased to below the limit of detection, this pre-enrichment broth was used to determine the presence/absence of *Salmonella* in 10 g, according to ISO 6579 method [16].

In preliminary studies the *Salmonella* count was carried out on TSA and XLD agar. The results showed that there was no significant difference (p>0.05) between TSA and XLD agar performance. Therefore, to avoid the interference of the background microflora in *Salmonella* determination throughout the storage time, XLD agar was used for all the subsequent experiments.

### Water activity and chemical composition

Water activity was measured before inoculation and at each sampling in duplicate at 25 ± 1°C with a water activity meter—hygrometer (Aqualab CX2, Decagon Device, Pullman, WA).

The chemical composition of peanuts and peanut confectionary products was carried out in triplicate using uninoculated samples at the end of the study. Moisture content was determined in an oven at 105 ± 1°C to constant weight [17]. The Kjeldahl method was used to estimate the nitrogen content and the result was converted to protein percentage by using the conversion factor 5.76 [18]. The fat content was determined according to the method of Bligh and Dyer [19]. Ash analysis was carried out in muffle at 550°C for 8 h [17]. Carbohydrate content was calculated by the difference between total dry matter and contents of ash, fat and protein, and the results were expressed as percent dry weight.

### Model fitting and statistical analysis

For each sample and inoculum level a survival curve for the *Salmonella* was estimated using the Weibull model [20] given by

$$log\left(\frac{N_{t}}{N_{0}}\right)=-{\left(\frac{t}{\delta }\right)}^{\beta },\mathrm{for}\;t>0$$ (1)

where t is the time (days), N_t_ is the population at time t (cfu/g), N_0_ is the population at time 0 (cfu/g), δ is a parameter representing the time required for first decimal reduction (days) and *β* is a parameter that defines the shape of the survival curve (dimensionless). Mean sum of squared error (MSE) and regression coefficient (R^2^) were used to access appropriateness of fit. T_3d_ is the time in days to reach three decimal reductions and was also estimated based on the Weibull model.

The likelihood ratio test [21] was used to compare the survival curves, two by two, between samples at the same inoculum level and the survival curves between different inoculum levels for the same sample. The goal of the likelihood ratio test is to compare the goodness of fit of two models, the null and the alternative were used. When the survival curves of two different samples are compared, the null model is the one that fits a common curve for both samples and the alternative model is the one that fits jointly distinct curves for the two different samples. For the multiple comparison tests, the p-values obtained were adjusted applying the fdr (false discovery rate) [22]. These analyses were performed using the R software (R Core Team, 2017) and the library nlme [21].

The decline rate was analyzed with one-way analysis of variance (ANOVA) and Tukey tests to determine whether there were significant differences (p<0.05). SAS software (version 9.4, SAS Institute, Cary, NC) was used.

## Results

### Water activity and chemical composition

**Fig 1:**
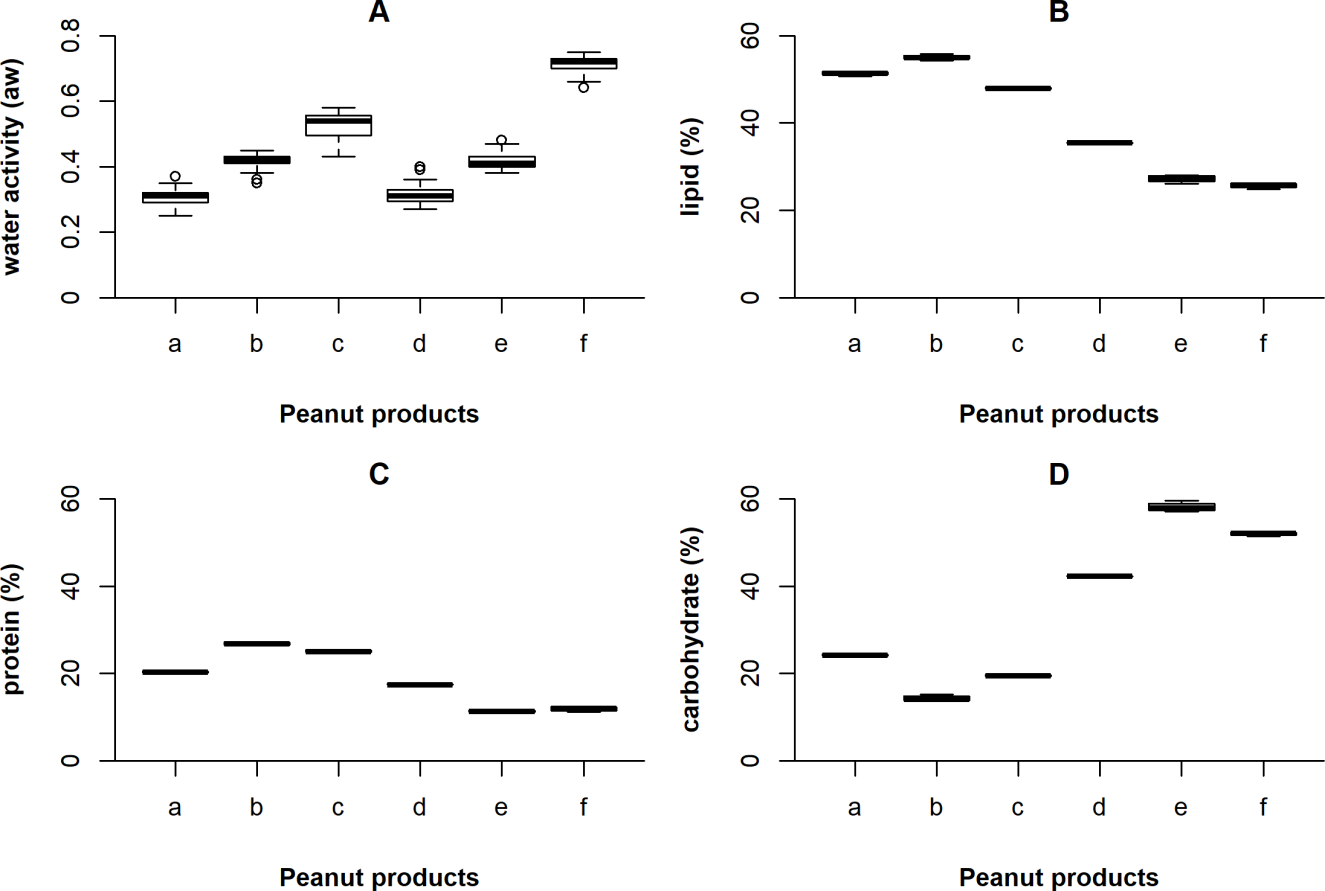
Box-whisker plot of the water activity (A), lipid content (B), protein content (C) and carbohydrate content (D) of raw in shell peanuts (a), roasted peanuts (b), unblanched peanut kernels (c), peanut brittle (d), *paçoca* (e) and *pé-de-moça* (f). Maximum and minimum are denoted by whiskers, 75^th^ and 25^th^ percentile are denoted by box, and median is the line inside the box.

The results of a_w_ and chemical composition of the peanut-based products are shown in Fig 1. Some products showed similar a_w_, but they differ in the composition (raw in-shell peanuts and peanut brittle; roasted peanuts and *paçoca*). There was no significant difference (p≥0.05) between the a_w_ values of uninoculated and inoculated samples throughout the storage time. The variations occurred mainly due to the opening of the microbiological incubator and the desiccators to collect samples for *Salmonella* enumeration. The media a_w_ value was 0.31 ± 0.02 for raw peanuts in-shell, 0.41 ± 0.02 for roasted peanuts, 0.53 ± 0.04 for unblanched peanut kernels, 0.32 ± 0.02 for peanut brittle, 0.41 ± 0.02 for *paçoca* and 0.69 ± 0.05 for *pé-de-moça* (Fig 1A). Fat content ranged between 25.68 and 55.01% (Fig 1B), protein between 11.35 and 26.83% (Fig 1C), and carbohydrate from 14.29 to 58.14% (Fig 1D).

### Inoculum level

During most of the long-term storage, for the same type of product, no significant difference (p>0.05) was observed when comparing the *Salmonella* decline rates of both inoculum levels (3 and 6 log cfu/g). The exceptions were for raw in-shell peanuts at day 7 and peanut brittle and *paçoca* at days 7, 14 and 45, and 14, 21,150,180 and 240, respectively. For these analyses only data up to the point at which one sample decreased to below the limit of detection (1 cfu/g) were used.

### Fate of *Salmonella* during long-term storage of peanut-based products

**Fig 2:**
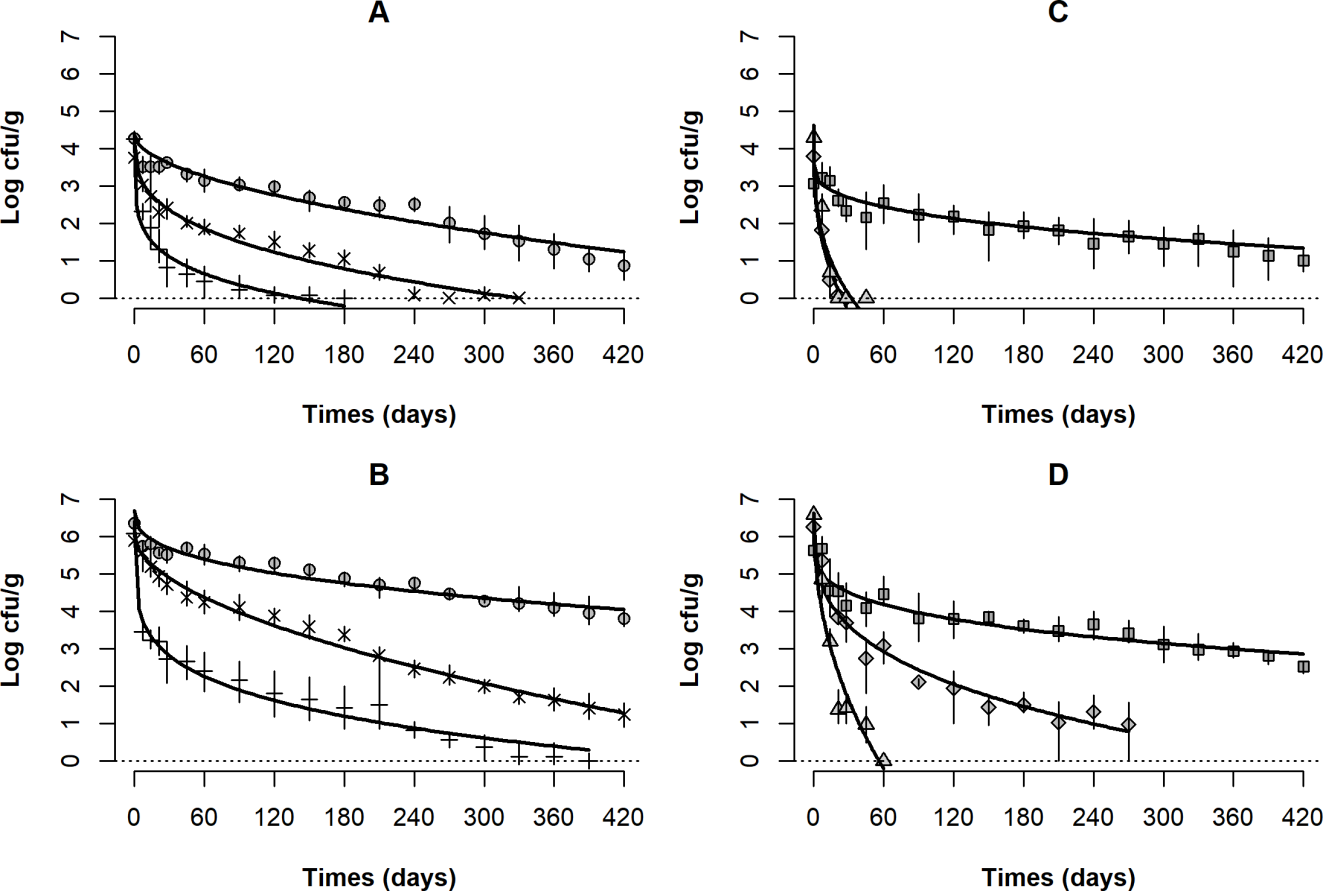
*Salmonella* Typhimurium ATCC 14028 survival curve artificially inoculated in peanut products stored at 28 ± 1° C. Low inoculum level 3 log cfu/ g (A and C). High inoculum level 6 log cfu/ g (B and D). Raw in-shell peanuts (+), roasted peanuts (●), unblanched peanut kernels (×), peanut brittle (♦), *paçoca* (■), *pé-de-moça* (▲).The dotted line represents the limit of detection for *Salmonella* of 0 log cfu/ g or 1 cfu/ g.

Fig 2 shows the reduction of *Salmonella* artificially inoculated in different peanut products during storage for 420 days at 28 ± 1°C. In experiment 1, after 28 days of storage, reductions of 0.7, 1.5 and 3.4 log cfu/g were obtained for roasted peanuts, unblanched peanut kernels and raw peanuts in-shell, respectively. After that, raw in-shell peanuts followed the trend of greater loss of *Salmonella* viability. From 240 days onwards, the pathogen was only recovered in this product by enrichment of 10-g samples (Table 1). The same condition was verified for unblanched peanut kernels after 330 days. At the end of the storage time, the lowest reduction in *Salmonella* counts was found in roasted peanuts which was 3.4 log cfu/g (Fig 2A). In experiment 2 (high inoculum level), reduction of 1-log was observed after less than 7 days in raw in-shell peanuts, 21 days in unblanched peanut kernels and 120 days in roasted peanuts. After 420 days, reductions of 2.6, 4.6 and 6.1 log cfu/g were observed for the roasted peanuts, unblanched peanut kernels and raw peanuts in-shell, respectively (Fig 2B).

**Table 1:** Presence of *Salmonella* Typhimurium ATCC 14028 in peanut products with counts below the limit of detection during a 420-days storage period at 28 ± 1° C.

Inoculum level (log cfu/g)	Sample	Storage (days)																	
		7	14	21	28	45	60	90	120	150	180	210	240	270	300	330	360	390	420
3.0	Raw in-shell peanuts						1 (1)^a^	2 (2)	1 (1)	3 (3)	4 (3)	4 (3)	4 (4)	4 (4)	4 (4)	4 (4)	4 (1)	4 (4)	4 (4)
	Roasted peanuts																		
	Unblanched peanut kernels												1 (1)	1 (1)	3 (3)	4 (4)	4 (4)	4 (4)	4 (4)
	Peanut brittle		2 (2)	1 (1)	3 (3)	2 (2)	4 (4)	4 (3)	4 (4)	4 (3)	4 (2)	4 (2)	4 (2)	4 (2)	4 (2)	4 (2)	4 (2)	4 (2)	4 (2)
	*Paçoca*																		
	*Pé-de-moça*		1 (1)	4 (4)	4 (3)	4 (2)	4 (1)	4 (1)	4 (0)	4 (2)	4 (0)	4 (0)	4 (0)	4 (0)	4 (0)	4 (0)	4 (0)	4 (0)	4 (0)
6.0	Raw in-shell peanuts														1 (1)	2 (2)	3 (3)	4 (4)	4 (4)
	Roasted peanuts																		
	Unblanched peanut kernels																		
	Peanut brittle								1 (1)			1(1)		1 (1)	1 (1)	1 (1)	1 (0)	1 (0)	2 (1)
	*Paçoca*																		
	*Pé-de-moça*						3 (3)	4 (4)	4(4)	4 (4)	4 (3)	4 (3)	4 (4)	4 (1)	4 (2)	4 (2)	4 (1)	4 (2)	4 (2)

^a^ Number of samples below the limit of detection (number of positive samples in 10 g after enrichment). Limit of detection in plate count method: 1 cfu/g.

In regard to confectionary products, in experiment 1, counts of *Salmonella* decreased sharply in peanut brittle and *pé-de-moça*, and were significantly lower than counts obtained in *paçoca*. *Salmonella* population approached the limit of detection (1 cfu/ g) after 21 days in *pé-de-moça* and 60 days in peanut brittle. From 180 days onwards, the pathogen could not be recovered by enrichment from any of the four 10g-samples analyzed for *pé-de-moça* (Table 1). At the end of the storage, only 50% of the sample of peanut brittle had viable cells after enrichment. Meanwhile, in *paçoca*, *Salmonella* population remained above the limit of detection throughout the study (Fig 2C). In experiment 2, *pé-de-moça* showed the greatest reduction, with counts below the limit of detection from the 60th day. However, the pathogen remained viable in at least 50% of the samples of this product until 420 days. *Paçoca* had the lowest *Salmonella* rate of decline: 1.1 and 3.1 log cfu/ g after 14 and 420 days, respectively. After these same periods, reductions of 1.6 and 5.8 log cfu/g were observed in peanut brittle (Fig 2D).

### Weibull model

The Weibull model was used to describe the experimental data. Data were included up to the point the samples fell below the limit of detection (1 cfu/g, minimum of six points). In most of the cases the R^2^ values obtained were higher than 0.9. The lowest R^2^ value was observed for *paçoca* at high inoculum level (0.81) (Table 2). This product had the highest standard deviation among its replicates. The highest MSE values were verified for *pé-de-moça*, 0.29 and 0.34. In general, the Weibull model fitted the data well over long time frames. The model only underestimates δ (time required for reduction of 1 log) for unblanched peanuts and *paçoca*, and roasted peanuts and peanut brittle at low and high inoculum levels, respectively. The *β* value, another parameter of the Weibull model, which defines the shape of the survival curve, ranged from 0.19 to 0.53 at the low inoculum level and from 0.21 to 0.54 at the high inoculum level. The parameter δ was also used to estimate T_3d_ values (time required to reduce 3 log of *Salmonella*). It ranged from 8 to 922 days, being influenced by the a_w_ of the samples.

**Table 2:** Parameters of the Weibull model fitted for *Salmonella* Typhimurium ATCC 14028 survival in peanut-based products with two inoculum levels during a 420 days storage period at 28 ± 1°C.

Inoculum level (log cfu/g)	Sample			Weibull model				
		δ (days)^a^	SE δ^b^	β^c^	SE β^d^	T_3d_^e^	R^2^ ^f^	MSE^g^
3.0	Raw in-shell peanuts	0.06	0.03	0.19	0.01	19.65	0.94	0.10
	Roasted peanuts	49.75	6.31	0.53	0.03	388.31	0.92	0.08
	Unblanched peanut kernels	4.91	0.83	0.34	0.02	122.06	0.94	0.08
	Peanut brittle	0.40	0.29	0.36	0.07	8.51	0.93	0.20
	*Paçoca*	35.71	11.33	0.34	0.02	922.24	0.87	0.08
	*Pé-de-moça*	0.23	0.19	0.31	0.05	8.05	0.90	0.29
6.0	Raw in-shell peanuts	0.06	0.02	0.21	0.01	12.79	0.95	0.13
	Roasted peanuts	29.96	4.11	0.37	0.02	592.33	0.89	0.06
	Unblanched peanut kernels	23.74	2.01	0.54	0.02	180.10	0.97	0.07
	Peanut brittle	0.80	0.24	0.30	0.02	30.09	0.92	0.22
	*Paçoca*	7.24	2.13	0.28	0.02	356.50	0.81	0.15
	*Pé-de-moça*	0.84	0.30	0.45	0.04	9.66	0.93	0.34

^a^ time required for first decimal reduction (days)

^b^ standard error of δ value

^c^ fitting parameter that defines the shape of the curve

^d^ standard error of β value

^e^ time required for three decimal reductions

^f^ regression coefficient

^g^ mean sum of square error.

## Discussion

*Salmonella* outbreaks linked to peanut based products have been reported in the last decades [1–7]. The contamination can occur at any point along the supply chain. In our study *Salmonella* survivability in raw peanuts in-shell, roasted peanuts, unblanched peanut kernels and three peanut confectionary products widely consumed in Brazil (peanut brittle, *paçoca* and *pé-de-moça*) was evaluated. All the analyzed products, with the exception of raw peanuts in-shell, are classified as ready-to-eat food (RTE). Storage was performed at 28 ± 1°C for 420 days. The temperature chosen for the study was based on meteorological data indicating that this is the average temperature of the spring and summer months in most of the country [23].

The presence of water is essential for bacterial growth. The reduction of a_w_ in the environment triggers protection mechanisms such as osmoregulation, which allows the cell to balance its internal composition with the external environment [24]. Water activity (a_w_) was the main factor that influenced (p<0.05) the *S*. Typhimurium survival in peanuts and peanut confectionary products. The lowest reductions were seen in a_w_ around 0.40 (roasted peanuts and *paçoca*), followed by 0.54 (unblanched peanut kernels) and 0.30 (raw peanuts in-shell and peanut brittle). The greatest reductions were observed in the product with the highest a_w_ (0.68—*pé-de-moça*). A similar result was reported by Tamminga et al. [25], who obtained a higher resistance of *S*. Typhimurium in chocolate with aw 0.40 than 0.37 or 0.42. In a study involving whey protein stored for 168 days at 21°C, a significant difference was observed between a_w_ 0.41 and 0.53, but not between 0.34 and 0.41 or 0.34 and 0.53 [26]. Juven et al. [27] evaluated the survival of *S*. Montevideo in different products and found a greater reduction in the pathogen at a_w_ 0.75 when compared to 0.52 and 0.43.

The lipid, protein and carbohydrate content of the peanut based products also had influence on the behavior of *Salmonella*. Using the likelihood ratio test between products with similar a_w_, but with different chemical composition (peanut brittle vs. raw in-shell peanuts and *paçoca* vs. roasted peanuts) we verified a significant difference (p < 0.05) in the *Salmonella* survival curves. For products with a_w_ 0.3 the highest *Salmonella* resistance was observed in peanut brittle, which has great carbohydrate content. On the other hand, for a_w_ 0.4 *Salmonella* survived for longer in roasted peanuts, which show higher fat content. In other words, a predominance impact of a specific chemical component (lipid, protein or carbohydrate) on the pathogen survival was not observed. According to Li et al [28] microenvironments present in the food matrix can heterogeneously influence the survival of *Salmonella* even in food with the same chemical composition or a_w_. He et al [29] evaluated *Salmonella* survival in peanut butter with the same a_w_ (0.40) and different fat and carbohydrate percentage. A greater survival was obtained in the samples with higher carbohydrate and less fat content. Other authors verified a significant influence of a_w_ on the first log-decimal reduction (log δ), whereas no significant differences were detected between products with different fat content [30]. *S*. Typhimurium inoculated in peanut butter with the same percentage of fat, but with different a_w_ (0.30 and 0.60) was recovered only in the product with the lowest a_w_ [31]. It is known that fat content directly affects cell moisture [32] and high sugar concentration increases heat resistance due to decreased a_w_ [33]. Nonetheless, the interaction among food macromolecules and bacteria resistance in desiccation stress condition remains to be elucidated.

No difference was observed in the rate of decline of *Salmonella* inoculated at two inoculum levels on four out of six peanut products analyzed. It corroborates with other reports that did not obtain a difference in *Salmonella* reduction using different inoculum levels (from 1 to 10 log cfu/g) during short or long storage of almonds [9, 34], walnut kernels [35] and peanut kernels [14]. In contrast, greater declines were observed using low inoculum level (3 log) on pecan halves and pieces [36].

The storage curves (Fig 2A–2D) show a non-linear kinetic decline of *Salmonella* population, with a fast loss of viability in the first two weeks, followed by a long-term phase of very slow decline. It can be more clearly observed at the high inoculum level experiment (Fig 2B and 2D). A non-linear kinetic death was also noted in different kinds of nuts and confectionary products [35–38]. In almonds inoculated with *S*. Enteritidis PT30, a reduction of 1.1 log cfu/ g was observed in the first 59 days at 35°C, whereas no reduction was noted between 59 and 171 days [34]. Park et al. [39] reported reductions of *Salmonella* in peanut butter (a_w_ *ca*. 0.2) between 0.3 and 1.3 log cfu/ g after 14 days at 22°C. According to some reports a rapid decline in bacteria counts shortly after inoculation can be caused by osmotic shock. The remaining cells would be able to adapt to the inhospitable environment, creating conditions to persist for a long time in low moisture products [25, 40].

The shelf life declared on the manufacturers’ labels ranges between 180 and 360 days at room temperature. Considering the predicted data obtained using the Weibull model at the high inoculum level experiment, the time needed to reach three decimal reductions of *Salmonella* (T_3d_) would take 356.5 and 592.3 days in *paçoca* and roasted peanuts, respectively. On the other hand, for *pé-de-moça* it would happen after *ca*. 10 days at 28± 1°C. Intermediate values were found for the other samples: around 13 days for raw in-shell peanuts, 30 days for peanut brittle, and 180 days for unblanched peanut kernels (Table 2). In addition, the data of presence/absence in 10 g at both inoculum levels showed that *Salmonella* viability extended beyond the shelf life of the products. The only exception was observed for *pé-de-moça* (a_w_ 0.68) when inoculated with the low inoculum level (Table 2). *Salmonella* was also recovered from shelled almonds after 550 days [34], and for at least 365 days on pecans, walnut kernels, almonds, pistachios and peanuts [35, 36, 9, 14]. On the other hand, a lower viability of *Salmonella* was reported by other authors in peanut butter for 6 weeks at 21°C [37]. Our results can cause concerns from a public health point of view, since the presence of *Salmonella* even at low levels (1 cfu/g) over the shelf life of these products could lead to illness [5,6].

In conclusion, the long-term *Salmonella* survivability in peanuts and peanut-based products was influenced by the water activity and the chemical composition. The lowest reductions were verified in roasted peanuts and *paçoca* (a_w_≈0.4). These data can be used for risk assessment studies on *Salmonella* in peanuts and peanut confectionary products. Nevertheless, further studies are needed to better understand the mechanisms involved in the resistance of *Salmonella* to dryness.

## Supporting information

S1 Table Commercial information about the analyzed samples. (DOCX)

S2 Table Chemical composition of the peanut products analyzed. (DOCX)

S3 Table Salmonella count in peanut confectionary product inoculated with low inoculum level and stored for 420 days. (DOCX)

S4 Table Salmonella count in peanut confectionary product inoculated with high inoculum level and stored for 420 days. (DOCX)

S5 Table Salmonella count in peanuts inoculated with low inoculum level and stored for 420 days. (DOCX)

S6 Table Salmonella count in peanuts inoculated with high inoculum level and stored for 420 days. (DOCX)

S7 Table Water activity (mean value of four samples) of the peanut products inoculated with tow inoculum levels and stored for 420 days. (DOCX)
