# Supplementary material for: Long-term kinetics of Salmonella Typhimurium ATCC 14028 survival on peanuts and peanut confectionery products
Source: PLoS One. 2018 Feb 5;13(2):e0192457. doi: 10.1371/journal.pone.0192457 (PMC5798841; doi:10.1371/journal.pone.0192457)
Supplement: S1 Table — (DOCX) [file pone.0192457.s001.docx]

S1 Table. Commercial information about the analyzed samples.

| **Product** | **Brand** | **Lot number** | **Expiration date** |
| --- | --- | --- | --- |
| Peanut brittle | Puro sabor | Not declared | 20/04/2015, 12/10/2015 |
| *Paçoca* | Dhole | 010 | 10/06/2014 |
|  | New Fly | 009 | 03/11/2016 |
| *Pé-de-moça* | Clamel | 0426, 0429 | 08/06/2015, 30/10/2015 |
| Raw in-shell peanuts | Amendupã | 2605 | 23/11/2015 |
| Roasted peanuts | Santa Helena | L1325M02 | 13/11/2015 |
| Unblanched peanut kernels | Yoki | B2L:B6JA 11:14 | 29/05/2016 |
