# Supplementary material for: Long-term kinetics of Salmonella Typhimurium ATCC 14028 survival on peanuts and peanut confectionery products
Source: PLoS One. 2018 Feb 5;13(2):e0192457. doi: 10.1371/journal.pone.0192457 (PMC5798841; doi:10.1371/journal.pone.0192457)
Supplement: S2 Table — (DOCX) [file pone.0192457.s002.docx]

S2 Table. Chemical composition of the peanut products analyzed.

| Product | Sample | Moisture (%) | Lipid (%) | Protein (%) | Ash (%) | Carbohydrate (%) |
| --- | --- | --- | --- | --- | --- | --- |
| Peanut brittle | 1 | 3,46 | 35,41 | 17,59 | 1,50 | 42,05 |
|  | 2 | 3,27 | 35,59 | 17,53 | 1,25 | 42,37 |
|  | 3 | 3,51 | 35,34 | 17,29 | 1,56 | 42,31 |
| *Paçoca* | 1 | 1,64 | 27,71 | 11,44 | 1,54 | 57,67 |
|  | 2 | 1,65 | 27,97 | 11,59 | 1,71 | 57,08 |
|  | 3 | 1,51 | 26,05 | 11,02 | 1,75 | 59,67 |
| *Pé-de-moça* | 1 | 8,96 | 26,14 | 11,16 | 1,56 | 52,17 |
|  | 2 | 8,73 | 26,01 | 12,31 | 1,52 | 51,43 |
|  | 3 | 8,95 | 24,87 | 12,24 | 1,61 | 52,34 |
| Roasted peanuts | 1 | 1,77 | 54,33 | 26,39 | 2,26 | 15,25 |
|  | 2 | 1,58 | 54,94 | 27,20 | 2,30 | 13,97 |
|  | 3 | 1,36 | 55,75 | 26,91 | 2,33 | 13,65 |
| Unblanched peanut kernels | 1 | 5,30 | 48,01 | 25,44 | 2,09 | 19,15 |
|  | 2 | 5,35 | 47,59 | 25,01 | 2,15 | 19,90 |
|  | 3 | 5,41 | 48,04 | 24,87 | 2,17 | 19,52 |
| Raw in-shell peanuts | 1 | 1,67 | 51,50 | 20,45 | 2,50 | 23,88 |
|  | 2 | 1,64 | 50,75 | 20,52 | 2,63 | 24,47 |
|  | 3 | 1,36 | 51,75 | 20,10 | 2,64 | 24,16 |
