# Supplementary material for: Long-term kinetics of Salmonella Typhimurium ATCC 14028 survival on peanuts and peanut confectionery products
Source: PLoS One. 2018 Feb 5;13(2):e0192457. doi: 10.1371/journal.pone.0192457 (PMC5798841; doi:10.1371/journal.pone.0192457)
Supplement: S3 Table — (DOCX) [file pone.0192457.s003.docx]

S3 Table. *Salmonella* count in peanut confectionary product inoculated with low inoculum level and stored for 420 days.

| Time (days) | *Salmonella* count (log cfu/g) | | | | | | | | | | | | | | | | | | |  |
| --- | --- | --- | --- | --- | --- | --- | --- | --- | --- | --- | --- | --- | --- | --- | --- | --- | --- | --- | --- | --- |
|  |  | Peanut brittle | | |  |  |  |  |  | *Paçoca* | |  |  |  |  |  | *Pé-de-moça* | | |  |
|  | R1 | R2 | R3 | R4 | mean | SD |  | R1 | R2 | R3 | R4 | mean | SD |  | R1 | R2 | R3 | R4 | mean | SD |
| 0 | 3,56 | 3,26 | 3,98 | 4,37 | 3,79 | 0,49 |  | 3,46 | 2,70 | 3,08 | 3,00 | 3,06 | 0,31 |  | 4,62 | 4,61 | 4,02 | 3,92 | 4,29 | 0,37 |
| 7 | 1,82 | 1,82 | 1,85 | 1,78 | 1,82 | 0,03 |  | 3,18 | 3,00 | 3,63 | 3,04 | 3,21 | 0,29 |  | 2,70 | 2,78 | 2,30 | 2,00 | 2,45 | 0,36 |
| 14 | 1,00 | 0,00 | 0,00 | 0,90 | 0,48 | 0,55 |  | 3,00 | 2,90 | 3,51 | 3,15 | 3,14 | 0,27 |  | 1,00 | 0,00 | 1,08 | 0,70 | 0,70 | 0,49 |
| 21 | 0,00 | 0,00 | 0,00 | 0,30 | 0,08 | 0,15 |  | 2,49 | 2,45 | 2,92 | 2,58 | 2,61 | 0,21 |  | 0,00 | 0,00 | 0,00 | 0,00 | 0,00 | 0,00 |
| 28 | 0,00 | 0,00 | 0,00 | 0,00 | 0,00 | 0,00 |  | 2,20 | 2,04 | 2,43 | 2,67 | 2,34 | 0,27 |  | 0,00 | 0,00 | 0,00 | 0,00 | 0,00 | 0,00 |
| 45 | 0,00 | 0,00 | 0,48 | 0,00 | 0,12 | 0,24 |  | 1,78 | 1,30 | 2,71 | 2,84 | 2,16 | 0,74 |  | 0,00 | 0,00 | 0,00 | 0,00 | 0,00 | 0,00 |
| 60 | 0,00 | 0,00 | 0,00 | 0,00 | 0,00 | 0,00 |  | 2,30 | 2,00 | 3,02 | 2,85 | 2,54 | 0,47 |  | 0,00 | 0,00 | 0,00 | 0,00 | 0,00 | 0,00 |
| 90 | 0,00 | 0,00 | 0,00 | 0,00 | 0,00 | 0,00 |  | 2,08 | 1,49 | 2,78 | 2,56 | 2,23 | 0,57 |  | 0,00 | 0,00 | 0,00 | 0,00 | 0,00 | 0,00 |
| 120 | 0,00 | 0,00 | 0,00 | 0,00 | 0,00 | 0,00 |  | 2,15 | 1,70 | 2,48 | 2,38 | 2,18 | 0,35 |  | 0,00 | 0,00 | 0,00 | 0,00 | 0,00 | 0,00 |
| 150 | 0,00 | 0,00 | 0,00 | 0,00 | 0,00 | 0,00 |  | 1,00 | 1,82 | 2,30 | 2,15 | 1,82 | 0,58 |  | 0,00 | 0,00 | 0,00 | 0,00 | 0,00 | 0,00 |
| 180 | 0,00 | 0,00 | 0,00 | 0,00 | 0,00 | 0,00 |  | 1,63 | 1,59 | 2,30 | 2,18 | 1,93 | 0,37 |  | 0,00 | 0,00 | 0,00 | 0,00 | 0,00 | 0,00 |
| 210 | 0,00 | 0,00 | 0,00 | 0,00 | 0,00 | 0,00 |  | 1,66 | 1,43 | 2,15 | 2,00 | 1,81 | 0,33 |  | 0,00 | 0,00 | 0,00 | 0,00 | 0,00 | 0,00 |
| 240 | 0,00 | 0,00 | 0,00 | 0,00 | 0,00 | 0,00 |  | 1,08 | 0,78 | 2,13 | 1,81 | 1,45 | 0,63 |  | 0,00 | 0,00 | 0,00 | 0,00 | 0,00 | 0,00 |
| 270 | 0,00 | 0,00 | 0,00 | 0,00 | 0,00 | 0,00 |  | 1,51 | 1,18 | 2,07 | 1,83 | 1,65 | 0,39 |  | 0,00 | 0,00 | 0,00 | 0,00 | 0,00 | 0,00 |
| 300 | 0,00 | 0,00 | 0,00 | 0,00 | 0,00 | 0,00 |  | 1,18 | 0,85 | 1,85 | 1,90 | 1,45 | 0,51 |  | 0,00 | 0,00 | 0,00 | 0,00 | 0,00 | 0,00 |
| 330 | 0,00 | 0,00 | 0,00 | 0,00 | 0,00 | 0,00 |  | 0,85 | ** | 1,95 | 1,95 | 1,58 | 0,64 |  | 0,00 | 0,00 | 0,00 | 0,00 | 0,00 | 0,00 |
| 360 | 0,00 | 0,00 | 0,00 | 0,00 | 0,00 | 0,00 |  | 1,08 | 0,30 | 1,81 | 1,79 | 1,25 | 0,72 |  | 0,00 | 0,00 | 0,00 | 0,00 | 0,00 | 0,00 |
| 390 | 0,00 | 0,00 | 0,00 | 0,00 | 0,00 | 0,00 |  | 0,85 | 0,48 | 1,60 | 1,58 | 1,13 | 0,56 |  | 0,00 | 0,00 | 0,00 | 0,00 | 0,00 | 0,00 |
| 420 | 0,00 | 0,00 | 0,00 | 0,00 | 0,00 | 0,00 |  | 0,70 | 0,70 | 1,28 | 1,34 | 1,01 | 0,35 |  | 0,00 | 0,00 | 0,00 | 0,00 | 0,00 | 0,00 |
