# Supplementary material for: Long-term kinetics of Salmonella Typhimurium ATCC 14028 survival on peanuts and peanut confectionery products
Source: PLoS One. 2018 Feb 5;13(2):e0192457. doi: 10.1371/journal.pone.0192457 (PMC5798841; doi:10.1371/journal.pone.0192457)
Supplement: S4 Table — (DOCX) [file pone.0192457.s004.docx]

S4 Table. *Salmonella* count in peanut confectionary product inoculated with high inoculum level and stored for 420 days.

| Time (days) | *Salmonella* count (log cfu/g) | | | | | | | | | | | | | | | | | | | |
| --- | --- | --- | --- | --- | --- | --- | --- | --- | --- | --- | --- | --- | --- | --- | --- | --- | --- | --- | --- | --- |
|  |  | Peanut brittle | | |  |  |  |  |  | *Paçoca* | |  |  |  |  |  | *Pé-de-moça* | | |  |
|  | R1 | R2 | R3 | R4 | mean | SD |  | R1 | R2 | R3 | R4 | mean | SD |  | R1 | R2 | R3 | R4 | mean | SD |
| 0 | 5,88 | 6,30 | 6,62 | 6,23 | 6,26 | 0,30 |  | 5,74 | 5,43 | 5,61 | 5,74 | 5,63 | 0,15 |  | 6,61 | 6,61 | 6,51 | 6,63 | 6,59 | 0,05 |
| 7 | 5,36 | 5,87 | 5,85 | 4,36 | 5,36 | 0,71 |  | 5,73 | 5,20 | 6,00 | 5,79 | 5,68 | 0,34 |  | 5,08 | 5,04 | 4,68 | 4,64 | 4,86 | 0,23 |
| 14 | 4,41 | 4,85 | 5,04 | 4,19 | 4,62 | 0,39 |  | 5,40 | 4,90 | 4,03 | 3,86 | 4,55 | 0,73 |  | 3,23 | 3,00 | 3,54 | 3,04 | 3,20 | 0,25 |
| 21 | 3,70 | 4,20 | 3,76 | 3,71 | 3,84 | 0,24 |  | 4,36 | 4,20 | 5,04 | 4,57 | 4,54 | 0,36 |  | 1,00 | 1,90 | 1,30 | 1,30 | 1,38 | 0,38 |
| 28 | 3,18 | 3,81 | 4,11 | 3,68 | 3,70 | 0,39 |  | 4,57 | 4,76 | 3,63 | 3,68 | 4,16 | 0,59 |  | 1,30 | 1,00 | 1,78 | 1,60 | 1,42 | 0,34 |
| 45 | 1,81 | 3,04 | 3,18 | 2,94 | 2,74 | 0,63 |  | 3,96 | 3,60 | 4,29 | 4,52 | 4,09 | 0,40 |  | 0,78 | 0,48 | 1,45 | 1,20 | 0,98 | 0,43 |
| 60 | 2,60 | 3,45 | 3,28 | 3,00 | 3,08 | 0,37 |  | 4,46 | 4,15 | 4,32 | 4,93 | 4,47 | 0,33 |  | 0,00 | 0,00 | 0,00 | 0,00 | 0,00 | 0,00 |
| 90 | 2,00 | 2,04 | 2,20 | 2,19 | 2,11 | 0,10 |  | 3,20 | 3,23 | 4,33 | 4,49 | 3,81 | 0,69 |  | 0,00 | 0,00 | 0,00 | 0,00 | 0,00 | 0,00 |
| 120 | 1,00 | 2,41 | 2,33 | 2,03 | 1,94 | 0,65 |  | 3,41 | 3,28 | 4,23 | 4,27 | 3,80 | 0,53 |  | 0,00 | 0,00 | 0,00 | 0,00 | 0,00 | 0,00 |
| 150 | 0,95 | 1,57 | 1,46 | 1,76 | 1,44 | 0,35 |  | 3,79 | 3,66 | 3,95 | 4,00 | 3,85 | 0,16 |  | 0,00 | 0,00 | 0,00 | 0,00 | 0,00 | 0,00 |
| 180 | 1,36 | 1,30 | 1,52 | 1,84 | 1,51 | 0,24 |  | 3,64 | 3,42 | 3,62 | 3,79 | 3,62 | 0,15 |  | 0,00 | 0,00 | 0,00 | 0,00 | 0,00 | 0,00 |
| 210 | 0,00 | 1,30 | 1,23 | 1,58 | 1,03 | 0,70 |  | 3,42 | 3,20 | 3,43 | 3,85 | 3,48 | 0,27 |  | 0,00 | 0,00 | 0,00 | 0,00 | 0,00 | 0,00 |
| 240 | 1,70 | 0,95 | 0,85 | 1,76 | 1,32 | 0,48 |  | 3,71 | 3,25 | 3,67 | 4,01 | 3,66 | 0,31 |  | 0,00 | 0,00 | 0,00 | 0,00 | 0,00 | 0,00 |
| 270 | 0,00 | 1,56 | 1,04 | 1,32 | 0,98 | 0,69 |  | 3,16 | 3,24 | 3,51 | 3,76 | 3,42 | 0,27 |  | 0,00 | 0,00 | 0,00 | 0,00 | 0,00 | 0,00 |
| 300 | 0,00 | 0,85 | 0,90 | 1,00 | 0,69 | 0,46 |  | 2,63 | 2,85 | 3,38 | 3,60 | 3,12 | 0,45 |  | 0,00 | 0,00 | 0,00 | 0,00 | 0,00 | 0,00 |
| 330 | 0,00 | 1,08 | 1,00 | 0,78 | 0,72 | 0,49 |  | 2,85 | 2,70 | 2,96 | 3,41 | 2,98 | 0,31 |  | 0,00 | 0,00 | 0,00 | 0,00 | 0,00 | 0,00 |
| 360 | 0,00 | 1,18 | 1,00 | 0,48 | 0,67 | 0,53 |  | 2,83 | 2,76 | 2,99 | 3,16 | 2,94 | 0,18 |  | 0,00 | 0,00 | 0,00 | 0,00 | 0,00 | 0,00 |
| 390 | 0,00 | 1,11 | 1,04 | 0,00 | 0,54 | 0,62 |  | 2,93 | 2,58 | 2,78 | 2,95 | 2,81 | 0,17 |  | 0,00 | 0,00 | 0,00 | 0,00 | 0,00 | 0,00 |
| 420 | 0,00 | 0,90 | 0,85 | 0,00 | 0,44 | 0,51 |  | 2,64 | 2,34 | 2,53 | 2,60 | 2,53 | 0,13 |  | 0,00 | 0,00 | 0,00 | 0,00 | 0,00 | 0,00 |
