# Supplementary material for: Long-term kinetics of Salmonella Typhimurium ATCC 14028 survival on peanuts and peanut confectionery products
Source: PLoS One. 2018 Feb 5;13(2):e0192457. doi: 10.1371/journal.pone.0192457 (PMC5798841; doi:10.1371/journal.pone.0192457)
Supplement: S5 Table — (DOCX) [file pone.0192457.s005.docx]

S5 Table. *Salmonella* count in peanuts inoculated with low inoculum level and stored for 420 days.

|  |  |  |  |  |  |  |  |  |  | Sample | |  |  |  |  |  |  |  |  |  |
| --- | --- | --- | --- | --- | --- | --- | --- | --- | --- | --- | --- | --- | --- | --- | --- | --- | --- | --- | --- | --- |
|  |  | Roasted peanuts | | |  |  |  |  |  | *Unblanched peanut kernels* | | | | |  |  | *Raw in-shell peanuts* | | | |
| Time (days) | R1 | R2 | R3 | R4 | media | SD |  | R1 | R2 | R3 | R4 | media | SD |  | R1 | R2 | R3 | R4 | media | SD |
| 0 | 4,34 | 4,05 | 4,36 | 4,34 | 4,27 | 0,15 |  | 3,23 | 3,49 | 4,21 | 4,08 | 3,75 | 0,47 |  | 4,03 | 4,36 | 4,34 | 4,29 | 4,26 | 0,15 |
| 7 | 3,36 | 3,34 | 3,78 | 3,54 | 3,51 | 0,20 |  | 3,11 | 3,04 | 3,11 | 2,85 | 3,03 | 0,12 |  | 2,30 | 2,23 | 2,23 | 2,48 | 2,31 | 0,12 |
| 14 | 3,73 | 3,11 | 3,83 | 3,40 | 3,52 | 0,33 |  | 2,70 | 2,30 | 3,10 | 2,76 | 2,72 | 0,33 |  | 1,30 | 1,90 | 2,15 | 2,20 | 1,89 | 0,41 |
| 21 | 3,45 | 3,31 | 3,65 | 3,60 | 3,50 | 0,15 |  | 1,90 | 2,45 | 2,62 | 2,18 | 2,29 | 0,32 |  | 1,40 | 1,83 | 1,00 | 0,95 | 1,30 | 0,41 |
| 28 | 3,68 | 3,60 | 3,56 | 3,63 | 3,62 | 0,05 |  | 2,11 | 2,32 | 2,56 | 2,65 | 2,41 | 0,24 |  | 0,85 | 0,85 | 0,30 | 1,28 | 0,82 | 0,40 |
| 45 | 3,45 | 3,10 | 3,40 | 3,29 | 3,31 | 0,16 |  | 2,20 | 1,95 | 1,95 | 1,90 | 2,00 | 0,14 |  | 0,60 | 0,60 | 0,30 | 1,04 | 0,64 | 0,30 |
| 60 | 3,12 | 2,84 | 3,45 | 3,13 | 3,14 | 0,25 |  | 1,78 | 1,78 | 2,11 | 1,70 | 1,84 | 0,18 |  | 0,48 | 0,48 | 0,00 | 0,85 | 0,45 | 0,35 |
| 90 | 2,89 | 2,87 | 3,23 | 3,09 | 3,02 | 0,17 |  | 1,60 | 1,60 | 1,95 | 1,74 | 1,72 | 0,17 |  | 0,30 | 0,00 | 0,00 | 0,60 | 0,23 | 0,29 |
| 120 | 3,14 | 2,82 | 3,08 | 2,87 | 2,98 | 0,16 |  | 1,30 | 1,34 | 1,78 | 1,60 | 1,51 | 0,23 |  | 0,00 | 0,00 | 0,00 | 0,30 | 0,08 | 0,15 |
| 150 | 2,79 | 2,32 | 2,88 | 2,79 | 2,70 | 0,25 |  | 1,08 | 1,11 | 1,48 | 1,38 | 1,26 | 0,20 |  | 0,00 | 0,30 | 0,00 | 0,00 | 0,08 | 0,15 |
| 180 | 2,56 | 2,54 | 2,66 | 2,46 | 2,56 | 0,08 |  | 0,90 | 0,85 | 1,28 | 1,18 | 1,05 | 0,21 |  | 0,00 | 0,00 | 0,00 | 0,00 | 0,00 | 0,00 |
| 210 | 2,71 | 2,15 | 2,51 | 2,52 | 2,47 | 0,23 |  | 0,70 | 0,48 | 0,90 | 0,60 | 0,67 | 0,18 |  | 0,00 | 0,00 | 0,00 | 0,00 | 0,00 | 0,00 |
| 240 | 2,32 | 2,45 | 2,68 | 2,61 | 2,52 | 0,16 |  | 0,00 | 0,00 | 0,30 | 0,00 | 0,08 | 0,15 |  | 0,00 | 0,00 | 0,00 | 0,00 | 0,00 | 0,00 |
| 270 | 1,95 | 1,48 | 2,45 | 2,15 | 2,01 | 0,41 |  | 0,00 | 0,00 | 0,00 | 0,00 | 0,00 | 0,00 |  | 0,00 | 0,00 | 0,00 | 0,00 | 0,00 | 0,00 |
| 300 | 1,49 | 1,30 | 2,20 | 1,90 | 1,72 | 0,40 |  | 0,00 | 0,30 | 0,00 | 0,00 | 0,08 | 0,15 |  | 0,00 | 0,00 | 0,00 | 0,00 | 0,00 | 0,00 |
| 330 | 1,40 | 1,00 | 1,95 | 1,78 | 1,53 | 0,42 |  | 0,00 | 0,00 | 0,00 | 0,00 | 0,00 | 0,00 |  | 0,00 | 0,00 | 0,00 | 0,00 | 0,00 | 0,00 |
| 360 | 1,11 | 0,78 | 1,72 | 1,58 | 1,30 | 0,43 |  | 0,00 | 0,00 | 0,00 | 0,00 | 0,00 | 0,00 |  | 0,00 | 0,00 | 0,00 | 0,00 | 0,00 | 0,00 |
| 390 | 0,78 | 0,70 | 1,34 | 1,36 | 1,05 | 0,35 |  | 0,00 | 0,00 | 0,00 | 0,00 | 0,00 | 0,00 |  | 0,00 | 0,00 | 0,00 | 0,00 | 0,00 | 0,00 |
| 420 | 0,60 | 0,48 | 1,20 | 1,18 | 0,87 | 0,38 |  | 0,00 | 0,00 | 0,00 | 0,00 | 0,00 | 0,00 |  | 0,00 | 0,00 | 0,00 | 0,00 | 0,00 | 0,00 |
