# Supplementary material for: Long-term kinetics of Salmonella Typhimurium ATCC 14028 survival on peanuts and peanut confectionery products
Source: PLoS One. 2018 Feb 5;13(2):e0192457. doi: 10.1371/journal.pone.0192457 (PMC5798841; doi:10.1371/journal.pone.0192457)
Supplement: S6 Table — (DOCX) [file pone.0192457.s006.docx]

S6 Table. *Salmonella* count in peanuts inoculated with high inoculum level and stored for 420 days.

|  |  |  |  |  |  |  |  |  |  | Sample | |  |  |  |  |  |  |  |  |  |
| --- | --- | --- | --- | --- | --- | --- | --- | --- | --- | --- | --- | --- | --- | --- | --- | --- | --- | --- | --- | --- |
|  |  | Roasted peanuts | | |  |  |  |  |  | *Unblanched peanut kernels* | | | | |  |  | *Raw in-shell peanuts* | | | |
| Time (days) | R1 | R2 | R3 | R4 | media | SD |  | R1 | R2 | R3 | R4 | media | SD |  | R1 | R2 | R3 | R4 | media | SD |
| 0 | 6,69 | 6,41 | 6,24 | 6,11 | 6,55 | 0,20 |  | 5,72 | 5,97 | 5,83 | 6,03 | 5,89 | 0,14 |  | 5,98 | 6,20 | 5,75 | 6,40 | 6,08 | 0,28 |
| 7 | 5,89 | 5,63 | 5,72 | 5,72 | 5,10 | 2,42 |  | 5,06 | 5,74 | 5,88 | 5,61 | 5,57 | 0,36 |  | 3,36 | 3,26 | 3,57 | 3,62 | 3,45 | 0,17 |
| 14 | 5,98 | 5,40 | 6,01 | 5,84 | 5,06 | 1,33 |  | 4,92 | 5,26 | 5,56 | 5,04 | 5,20 | 0,28 |  | 3,12 | 3,08 | 3,18 | 3,51 | 3,22 | 0,20 |
| 21 | 5,64 | 5,51 | 5,66 | 5,48 | 4,90 | 1,78 |  | 4,88 | 5,12 | 5,05 | 4,67 | 4,93 | 0,20 |  | 3,59 | 2,83 | 3,28 | 3,06 | 3,19 | 0,32 |
| 28 | 5,41 | 5,30 | 5,72 | 5,65 | 4,75 | 1,48 |  | 4,56 | 5,02 | 4,85 | 4,46 | 4,72 | 0,26 |  | 2,74 | 2,08 | 3,20 | 2,90 | 2,73 | 0,47 |
| 45 | 5,85 | 5,61 | 5,60 | 5,70 | 4,84 | 1,69 |  | 4,18 | 4,36 | 4,81 | 4,15 | 4,38 | 0,30 |  | 2,51 | 2,18 | 3,09 | 2,87 | 2,66 | 0,40 |
| 60 | 5,48 | 5,25 | 5,64 | 5,79 | 4,76 | 1,53 |  | 3,93 | 4,26 | 4,56 | 4,23 | 4,25 | 0,26 |  | 2,08 | 1,85 | 2,76 | 2,91 | 2,40 | 0,51 |
| 90 | 5,28 | 5,07 | 5,41 | 5,48 | 4,68 | 1,59 |  | 3,76 | 4,15 | 4,45 | 4,08 | 4,11 | 0,28 |  | 1,79 | 1,56 | 2,61 | 2,67 | 2,16 | 0,57 |
| 120 | 5,33 | 5,06 | 5,45 | 5,35 | 4,59 | 1,50 |  | 3,63 | 3,98 | 4,09 | 3,85 | 3,89 | 0,20 |  | 1,40 | 1,18 | 2,26 | 2,41 | 1,81 | 0,61 |
| 150 | 5,24 | 4,94 | 5,13 | 5,12 | 4,51 | 1,51 |  | 3,26 | 3,65 | 3,90 | 3,54 | 3,59 | 0,27 |  | 1,20 | 1,08 | 2,07 | 2,24 | 1,65 | 0,59 |
| 180 | 5,05 | 4,67 | 4,81 | 5,01 | 4,33 | 1,41 |  | 3,30 | 3,41 | 3,60 | 3,19 | 3,38 | 0,17 |  | 0,95 | 0,85 | 1,89 | 2,00 | 1,42 | 0,61 |
| 210 | 4,72 | 4,35 | 4,85 | 4,91 | 4,10 | 1,35 |  | 2,23 | 3,05 | 2,98 | 3,01 | 2,82 | 0,39 |  | 0,85 | 1,18 | 1,54 | 2,41 | 1,50 | 0,67 |
| 240 | 4,90 | 4,62 | 4,73 | 4,78 | 4,12 | 1,39 |  | 2,69 | 2,60 | 2,34 | 2,23 | 2,47 | 0,22 |  | 0,70 | 1,00 | 0,78 | 0,85 | 0,83 | 0,13 |
| 270 | 4,52 | 4,43 | 4,60 | 4,31 | 4,00 | 1,30 |  | 2,57 | 2,30 | 2,04 | 2,04 | 2,24 | 0,25 |  | 0,48 | 0,70 | 0,48 | 0,60 | 0,57 | 0,11 |
| 300 | 4,23 | 4,30 | 4,31 | 4,27 | 3,79 | 1,24 |  | 2,18 | 1,98 | 2,09 | 1,81 | 2,02 | 0,16 |  | 0,30 | 0,00 | 0,70 | 0,48 | 0,37 | 0,30 |
| 330 | 4,66 | 4,18 | 4,01 | 4,02 | 3,74 | 1,26 |  | 1,78 | 1,70 | 1,85 | 1,52 | 1,71 | 0,14 |  | 0,00 | 0,00 | 0,48 | 0,00 | 0,12 | 0,24 |
| 360 | 4,51 | 4,07 | 3,92 | 3,88 | 3,60 | 1,17 |  | 1,96 | 1,54 | 1,68 | 1,34 | 1,63 | 0,26 |  | 0,00 | 0,00 | 0,00 | 0,48 | 0,12 | 0,24 |
| 390 | 4,41 | 3,95 | 3,78 | 3,65 | 3,49 | 1,16 |  | 1,81 | 1,20 | 1,54 | 1,11 | 1,42 | 0,32 |  | 0,00 | 0,00 | 0,00 | 0,00 | 0,00 | 0,00 |
| 420 | 4,08 | 3,60 | 3,91 | 3,67 | 3,29 | 1,06 |  | 1,55 | 1,23 | 1,30 | 0,90 | 1,25 | 0,27 |  | 0,00 | 0,00 | 0,00 | 0,00 | 0,00 | 0,00 |
