# Supplementary material for: Long-term kinetics of Salmonella Typhimurium ATCC 14028 survival on peanuts and peanut confectionery products
Source: PLoS One. 2018 Feb 5;13(2):e0192457. doi: 10.1371/journal.pone.0192457 (PMC5798841; doi:10.1371/journal.pone.0192457)
Supplement: S7 Table — (DOCX) [file pone.0192457.s007.docx]

S7 Table. Water activity (mean value of four samples) of the peanut products inoculated with tow inoculum levels and stored for 420 days.

|  |  |  |  | |  |  | Water activity (aw) | | | | | |  |  | | |  |  |  |
| --- | --- | --- | --- | --- | --- | --- | --- | --- | --- | --- | --- | --- | --- | --- | --- | --- | --- | --- | --- |
| Time (days) |  |  | low inoculum level | | |  |  | | | |  |  |  | high inoculum level | |  | | |  |
|  | peanut brittle | *paçoca* | *pé-de-moça* | roasted peanuts | | unblanched  peanut kernels | raw in-shell peanuts | | Peanut  brittle | | | | *paçoca* | *pé-de-moça* | roasted peanuts | unblanched peanut kernels | | | raw in-shell peanuts |
| 0 | 0,29 | 0,43 | 0,68 | 0,39 | | 0,53 | 0,28 |  | | 0,31 | | | 0,41 | 0,69 | 0,39 | 0,55 | | | 0,30 |
| 7 | 0,28 | 0,41 | 0,70 | 0,42 | | 0,56 | 0,29 |  | | 0,29 | | | 0,41 | 0,73 | 0,43 | 0,56 | | | 0,30 |
| 14 | 0,27 | 0,41 | 0,71 | 0,39 | | 0,55 | 0,28 |  | | 0,29 | | | 0,42 | 0,72 | 0,37 | 0,54 | | | 0,29 |
| 21 | 0,30 | 0,42 | 0,72 | 0,42 | | 0,55 | 0,27 |  | | 0,32 | | | 0,42 | 0,72 | 0,41 | 0,56 | | | 0,33 |
| 28 | 0,28 | 0,41 | 0,71 | 0,42 | | 0,54 | 0,29 |  | | 0,31 | | | 0,42 | 0,72 | 0,40 | 0,53 | | | 0,29 |
| 45 | 0,32 | 0,42 | 0,72 | 0,40 | | 0,49 | 0,31 |  | | 0,32 | | | 0,45 | 0,70 | 0,37 | 0,49 | | | 0,31 |
| 60 | 0,29 | 0,40 | 0,70 | 0,41 | | 0,51 | 0,31 |  | | 0,31 | | | 0,41 | 0,66 | 0,41 | 0,51 | | | 0,31 |
| 90 | 0,30 | 0,41 | 0,68 | 0,40 | | 0,45 | 0,31 |  | | 0,32 | | | 0,40 | 0,68 | 0,41 | 0,48 | | | 0,31 |
| 120 | 0,31 | 0,42 | 0,70 | 0,41 | | 0,43 | 0,32 |  | | 0,33 | | | 0,41 | 0,67 | 0,39 | 0,45 | | | 0,34 |
| 150 | 0,34 | 0,43 | 0,67 | 0,42 | | 0,49 | 0,33 |  | | 0,32 | | | 0,44 | 0,71 | 0,42 | 0,49 | | | 0,34 |
| 180 | 0,34 | 0,41 | 0,67 | 0,43 | | 0,54 | 0,33 |  | | 0,34 | | | 0,42 | 0,71 | 0,42 | 0,52 | | | 0,32 |
| 210 | 0,34 | 0,41 | 0,68 | 0,43 | | 0,55 | 0,33 |  | | 0,36 | | | 0,41 | 0,70 | 0,42 | 0,55 | | | 0,33 |
| 240 | 0,32 | 0,41 | 0,67 | 0,43 | | 0,56 | 0,35 |  | | 0,35 | | | 0,43 | 0,63 | 0,42 | 0,56 | | | 0,35 |
| 270 | 0,35 | 0,44 | 0,69 | 0,44 | | 0,54 | 0,33 |  | | 0,32 | | | 0,40 | 0,61 | 0,43 | 0,54 | | | 0,33 |
| 300 | 0,32 | 0,42 | 0,69 | 0,43 | | 0,52 | 0,33 |  | | 0,34 | | | 0,42 | 0,65 | 0,42 | 0,49 | | | 0,32 |
| 330 | 0,32 | 0,42 | 0,69 | 0,42 | | 0,56 | 0,32 |  | | 0,32 | | | 0,40 | 0,60 | 0,42 | 0,54 | | | 0,31 |
| 360 | 0,33 | 0,40 | 0,69 | 0,43 | | 0,56 | 0,36 |  | | 0,34 | | | 0,41 | 0,65 | 0,42 | 0,57 | | | 0,31 |
| 390 | 0,32 | 0,41 | 0,69 | 0,42 | | 0,55 | 0,30 |  | | 0,34 | | | 0,42 | 0,65 | 0,42 | 0,56 | | | 0,29 |
| 420 | 0,32 | 0,40 | 0,69 | 0,42 | | 0,55 | 0,31 |  | | 0,32 | | | 0,42 | 0,69 | 0,43 | 0,55 | | | 0,31 |
